# Clinical audit investigating the recognition of tardive dyskinesia in an acute inpatient setting

**DOI:** 10.1192/bjo.2021.303

**Published:** 2021-06-18

**Authors:** Daniel Romeu, Christiana Elisha-Aboh, Hamza Abid, Lauren Merry, Tariq Mahmood, Fiona Lacey

**Affiliations:** 1 Leeds and York Partnership NHS Foundation Trust, Leeds Institute of Health Sciences, University of Leeds; 2 Leeds and York Partnership NHS Foundation Trust; 3 Leeds Teaching Hospitals NHS Trust

## Abstract

**Aims:**

Tardive dyskinesia (TD) is a disabling extra-pyramidal side effect (EPSE) associated with long-term antipsychotic medication, with an incidence rate of 5% per year of typical antipsychotic exposure. The Abnormal Involuntary Movement Scale (AIMS) is a validated tool for screening for TD and its use is recommended every 3–6 months in those taking antipsychotics. Atypical antipsychotics present a lower risk and have contributed to complacency in monitoring and treatment. The primary aim of this audit was to establish whether AIMS was completed for all patients taking regular antipsychotic medication for three months or more. Secondary aims were to investigate whether patients were informed about EPSEs on initiation, titration and change of antipsychotics, and whether they were assessed for the emergence of side effects during subsequent clinical reviews.

**Method:**

This single-site audit examined the care of inpatients on Ward 4 of the Becklin Centre, a male working-age acute psychiatric ward, between 1st November 2020 and 31st January 2021. Patients aged 18–65 years who were prescribed regular antipsychotics were eligible for inclusion. Exclusion criteria included the presence of other neurological movement disorders. 50 patients were included. Data collection took place between 8th February and 6th March 2021; this involved reviewing patient records throughout their inpatient stay on Care Director, an electronic patient record system. Results were compiled using a pre-determined data collection tool and analysed using Microsoft Excel.

**Result:**

For 14 (28.0%) patients there was documented evidence of the provision of verbal information surrounding EPSEs during initiation or change of antipsychotics, and 12 (24.0%) received written or verbal information about wider side effects. For 19 (38.0%) there was a documented assessment of side effects during clinical review following the initiation or change of antipsychotic medication. Of the 33 patients who took antipsychotics for over three months, 3 (9.1%) received an AIMS assessment.

**Conclusion:**

An inadequate proportion of inpatients prescribed long-term antipsychotics were assessed for TD, likely due to a lack of awareness of the relevant guidance. A substantial number of patients were not informed about side effects, suggesting an element of medical paternalism. This study provides opportunity to improve practice by educating the medical workforce and raising awareness of TD symptoms amongst the wider team. Valbenazine is a new FDA-approved treatment for adults with tardive dyskinesia, representing a further avenue for management. Greater focus on patient involvement, and communication surrounding anticipated side effects, is likely to benefit compliance with treatment and improve the doctor-patient relationship.

